# Undernutrition was a prevalent clinical problem among older adult patients with heart failure in a hospital setting in Northwest Ethiopia

**DOI:** 10.3389/fnut.2022.962497

**Published:** 2022-12-01

**Authors:** Hussen Ahmed, Abilo Tadesse, Hailemaryam Alemu, Alula Abebe, Melaku Tadesse

**Affiliations:** Department of Internal Medicine, School of Medicine, College of Medicine and Health Sciences, University of Gondar, Gondar, Ethiopia

**Keywords:** heart failure, undernutrition, MNA-FL score, University of Gondar, Ethiopia

## Abstract

**Background:**

Undernutrition is a frequently noticed medical problem in patients with heart failure. It is caused by poor nutrient intake, malabsorption, systemic inflammation, neurohumoral activation, oxidative stress, and hypermetabolic state. Undernutrition results in a decrease in the quality of life and the survival rate in patients with heart failure. There is a paucity of documentation on undernutrition among patients with heart failure in sub-Saharan African countries. The study aimed to determine the magnitude and associated factors of undernutrition among older adult patients with heart failure in the hospital setting in Northwest Ethiopia.

**Methods:**

An institutional-based cross-sectional study was conducted at the University of Gondar Hospital, Northwest Ethiopia, between 1 June 2021 and 31 October 2021. A consecutive sampling method was used to recruit 262 study subjects. A Mini-nutritional assessment-full form (MNA-FL) Questionnaire was used to extract nutritional information among patients with heart failure. Patients with heart failure, who scored MNA-FL score <17, were declared to have undernutrition. The data were entered into EPI Info version 4.6.0.0 and then exported to SPSS version 26 for analysis. Explanatory variables associated with undernutrition in patients with heart failure were analyzed by applying a logistic regression model. A *P*-value of <0.05 was used to declare a significant association.

**Results:**

A total of 262 patients with heart failure were included in the study. The mean age (± SD) of the study subjects was 64.6 (± 9.2) years. Hypertensive heart disease (111/262, 42%) was the most common cause of heart failure. Hypertension was the frequently observed comorbid disease. Based on the MNA-FL score for nutritional status, 75 out of 262 (28.6%, 95% CI: 22.9–34.4%) were undernourished (MNA-FL < 17), while 124 out of 262 (47.3%, CI: 41.5–53.1%) were at risk of undernutrition (MNA-FL = 17–23.5). The remaining 63 out of 262 (24.1%, 95% CI: 18.2–29.8%) study subjects were well nourished (MNA-FL > 24). On a multivariate analysis, patients with severe heart failure (New York Heart Association (NYHA) functional class III/IV) (AOR = 4.287, CI: 2.012–9.134, *P*-value < 0.001), with a duration of illness of 3–5 years (AOR = 3.225, CI: 1.138–9.137, *P*-value = 0.028), with a duration of illness of >5 years (AOR = 4.349, CI: 1.592–11.879, *P*-value = 0.001), presence of comorbidities (AOR = 2.29, CI: 1.06–4.96, *P*-value = 0.036), who underwent treatment with loop diuretics (AOR = 2.983, CI: 1.407–6.326, *P*-value = 0.040), and who reside in a rural area (AOR = 5.119, CI: 2.481–10.560, *P*-value < 0.001) were at risk of developing undernutrition.

**Conclusion:**

Undernutrition was a significant clinical problem in older patients with heart failure. Nutritional interventions should be prioritized for patients with chronic and severe heart failure.

## Background

Heart failure (HF) is a clinical syndrome that results from structural or functional impairment of ventricular filling or ejection of the blood. It clinically presents with dyspnea and fatigue, which may limit exercise tolerance and fluid retention (1). Heart failure was estimated to affect 1–2% of the adult population worldwide (2). It is estimated that developing countries carry 80% of the global cardiovascular burden (3). Heart failure is frequently associated with malnutrition, termed cardiac wasting or cachexia. It is triggered by poor nutritional intake, malabsorption, systemic inflammation, neurohumoral activation, oxidative stress, and augmented catabolic process (4–7). Malnutrition in heart failure is associated with higher hospital readmission rates and increased mortality rates (7–12). Obese individuals with heart failure were paradoxically associated with improved cardiovascular outcomes and survival, which is referred to as the obesity paradox. However, one-third of elderly obese individuals had a nutritional deficiency and were at risk of adverse cardiovascular outcomes (4, 11, 13, 14). Nutritional interventions could potentially reduce hospital readmission rates and mortality in undernourished patients with heart failure (7, 11, 14–17). However, there is no accepted nutritional guideline for the management of malnutrition among patients with heart failure. There is a scarcity of data on heart failure-related malnutrition rates in sub-Saharan African countries. A single hospital-based study done in Ethiopia documented that more than three-fourths (78%) of patients with heart failure were undernourished (18). There are several techniques used to measure the nutritional status of patients with heart failure. The mini-nutritional assessment (MNA), the controlling nutritional status index (CONUT), the geriatric nutritional risk index (GNRI), the prognostic nutritional index (PNI), the malnutrition universal screening tool (MUST), and the subjective global assessment (SGA) were the frequently used nutritional assessment methods (19, 20). So far, there is no single-standard measuring tool to assess the nutritional status of patients with heart failure. However, MNA surpassed the other nutritional assessment tools in predicting hospital readmission rates, frailty, and mortality rates (7–9, 19, 21). In this study, the mini-nutritional assessment-full form (MNA-FL) nutritional assessment tool was used to determine the nutritional status of the study subjects. Identifying clinical predictors of undernutrition in patients with heart failure would assist in priority setting for nutritional intervention.

## Materials and methods

### Study settings and design

An institutional-based cross-sectional study was conducted at the Cardiac Clinic, Department of Internal Medicine, University of Gondar Hospital, between 1 June 2021 and 31 October 2021. The hospital is located in Northwest Ethiopia, which is 750 km away from the capital, Addis Ababa. The hospital served a catchment population of 7 million people. The hospital had all the major and minor clinical departments. Department of Internal Medicine was one of the major clinical departments. It had five general wards with 110 beds, one medical ICU with 12 beds, and five outpatient clinics. Other services in the department included multidrug resistant tuberculosis (MDR-TBC) service, dialysis service, endoscopy service, HIV/AIDS care, and chronic illness care. The Cardiac Clinic provided outpatient medical services for patients with cardiac diseases. The clinic was staffed with a cardiologist, internists, medical residents, medical practitioners, and unit nurses. The clinic was equipped with an Echo machine (B/W Digital Ultrasound Scanner, ARI Group, China) and an ECG machine (ECG 1200G, YSIP-155, Beijing, China).

### Study population and study subjects

#### Study population

Adult patients with heart failure were asked to return for follow-up at the Cardiac Clinic of the hospital.

#### Study subjects

Patients with heart failure aged 50 years or older were asked to return for follow-up at the Cardiac Clinic of the hospital during the study period. Relatively older adult patients with heart failure were recruited to keep measuring the consistency of the MNA-FL score.

#### Exclusion criteria

Patients with heart failure who were unable to give consent were excluded from the study.

#### Study variables

Dependent variable: Undernutrition (MNA-FL score < 17).

Independent variables are as follows: (1) sociodemographic characteristics—age, gender, residence, marital status, religion, and educational status; (2) clinical characteristics—cause of heart failure, duration of heart failure, severity of heart failure, comorbidities, and oral cardiac medication types.

#### Sample size and sampling procedures

The sample size was calculated based on single population proportion formula at a prevalence of undernutrition among patients with heart failure in Southwest Ethiopia was 78%, with a confidence interval of 95% and an assumed margin of error of 5% (14). A consecutive sampling method was used to recruit a sample size of 262 study subjects (22).

#### Data collection instruments and procedures

Data were collected through a structured questionnaire (MNA-FL). Patients were interviewed to obtain sociodemographic data and relevant medical and nutritional information. Weight (kg), height (m), mid-arm circumference (MAC) (cm), and calf circumference (cm) were measured and a focused clinical examination was carried out for each of the study subjects. Laboratory values were obtained from patients’ records. Echocardiography and ECG tests were carried out on all study subjects to determine the cause of heart failure and document the presence of arrhythmia, respectively.

#### Mini-nutritional assessment-full form score measurement

Nutritional status as undernutrition, at risk of undernutrition, and normal nutrition was declared by using the MNA-FL score. The MNA-FL score is a single and rapid nutrition assessment tool which was initially developed to assess the nutritional status of the elderly, but it was subsequently being used to assess the nutritional status of patients with chronic illnesses like patients with heart failure, malignancy, and chronic kidney disease. The MNA-FL score includes 18 items grouped into the following four categories: anthropometric assessment (BMI, weight loss, mid-arm, and calf circumferences); general assessment (lifestyle, medication, mobility, presence of depression/dementia, or pressure sores); dietary assessment (number of meals, food and fluid intake, and autonomy of feeding); and subjective assessment (self-perception of health and nutrition). Each answer has a numerical value and contributes to the final score, which has a maximum of 30, with threshold values of ≥24 for subjects who were well nourished, 17–23.5 for subjects at risk of undernutrition, and <17 for subjects who were undernourished (23).

#### Anthropometric measurement

Body mass index (BMI) was calculated as weight in kilogram divided by height in squared meters (kg/m^2^). The participant’s weight (kilogram) and height (meters) were measured according to the standard anthropometry procedures to determine the BMI. The adjusted dry weight was used in edematous patients (24). The MAC was measured in the non-dominant hand at a mid-point distance between the shoulder and the elbow. The calf circumference (CC) was measured at the widest circumference of the calf of the non-dominant leg with knee flexion at 90°. The MAC and CC were adjusted by subtracting 2 cm from the measured value in the edematous arm and leg, respectively (24).

#### Clinical procedures

Twelve-Lead ECG (ECG 1200G, YSIP-155, Beijing, China) was performed for all patients by a physician with a standardization of 1 mV = 10 mm and a paper speed of 25 mm/sec. Abnormal findings on the ECG were interpreted by a cardiologist.

Two-Dimensional Doppler Transthoracic Echocardio-graphy (B/W Digital Ultrasound Scanner, ARI Group, China) was performed for all patients by a cardiologist to determine abnormalities in ventricular ejection fraction, valve morphology, ventricular wall size and motion, and atrial and ventricular chamber dimensions.

#### Laboratory procedures

Venous blood samples were collected from patients in plain tubes and centrifuged at 2500 rpm for 15 min at room temperature to obtain serum. Mindray BS-480 (Shenzhen Mindray Bio-Medical Electronics Co., Ltd., China) clinical chemistry analyzer was used to determine serum glucose and creatinine values by enzymatic glucose oxidase and kinetic alkaline picrate method, respectively. While radioimmunoassay (RIA) technique (Roche, Switzerland) was used to determine thyroid function tests, and kits were from Beijing Isotope Nuclear Electronic Co., Beijing, China.

### Data analysis

Data were entered into and cleaned in EPI Info version 4.6.0.0 (EPI Info, Atlanta, USA) and transported to and analyzed in SPSS version 26 (SPSS Inc., Chicago, USA). Categorical variables were reported as frequencies (percentages) and continuous variables as the mean with standard deviation. The results were summarized using frequency, tables, and graphs. Risk factors for undernutrition were analyzed by applying a logistic regression model. The adequacy of the model was checked by using the Hosmer and Lameshow goodness-of-fit test. Those variables with a *P*-value of <0.25 in the bivariate analysis were exported to multivariate analysis. The results were presented as an odds ratio with 95% confidence interval. A *P*-value of <0.05 was used to declare a significant association.

### Ethical considerations

The research protocol complied with the Declaration of Helsinki, and ethical clearance was obtained from the Institutional Review Board (IRB) of the College of Medicine and Health Sciences, University of Gondar (02/08/2021, IRB No. 755/2021). Study subjects were recruited only after written informed consent was obtained. All data obtained were treated confidentially. Patients with heart failure were taken care of as per the recommendation of 2013 ACCF/AHA Guideline for the management of heart failure (1).

### Definition of terms

Undernutrition: A metabolic state resulting from the lack of intake or uptake of nutrition that leads to altered body composition (decreased fat-free mass) and body cell mass, leading to diminished physical and mental function and impaired clinical outcome from disease (25).

Heart failure: Symptoms and/or signs of heart failure caused by a structural and/or functional cardiac abnormality and corroborated by at least one of the following: elevated natriuretic peptide level or objective evidence of cardiogenic pulmonary or systemic congestion (26).

Hypertensive heart disease: Clinical symptoms and/or signs derived from left ventricular hypertrophy and dysfunction, myocardial ischemia, and rhythm abnormalities, all of them caused by the effects on the heart of chronically elevated blood pressure (27).

Ischemic heart disease: Presence of typical angina with angiography-confirmed coronary artery stenosis, documented prior myocardial infarction, or documented prior coronary artery revascularization (either PCI or CABG) (28).

Comorbidity: Any distinct additional entity that has existed or may occur during the clinical course of a patient who has the index disease under study (29).

Urban population: People living in urban areas as defined by National Statistical Offices (30).

Rural population: People living in rural areas as defined by National Statistical Offices (30).

## Results

### Sociodemographic characteristics of study participants

A total of 262 patients were included in this study. The mean age (± SD) of the study subjects was 64.6 (± 9.2) years. The majority (138/262, 53%) of the study participants were in the age range of 50–65 years, were women (154/262, 59%), and were rural residents (139/262, 53%) (Table 1).

**TABLE 1:** Socio-demographic characteristics of older adult patients with heart failure at Cardiac Clinic, University of Gondar hospital, June 01, 2021 to October 31, 2021 (No. 262).

Characteristics	Category	Frequency (No.)	Percentage (%)
Age	50–65 66–75 >75	138 78 46	52.7 29.8 17.5
Gender	Male Female	108 154	41.2 58.8
Marital status	Married Unmarried	167 95	63.7 36.3
Religion	Christian Muslim	240 22	91.6 8.4
Residence	Rural Urban	139 123	53.1 46.9
Educational status	Can’t read and write Read and write only Primary school	210 31 5	80.2 11.8 1.9
	Secondary school College and above	10 6	3.8 2.3

### Clinical characteristics of study subjects

**FIGURE 1:**
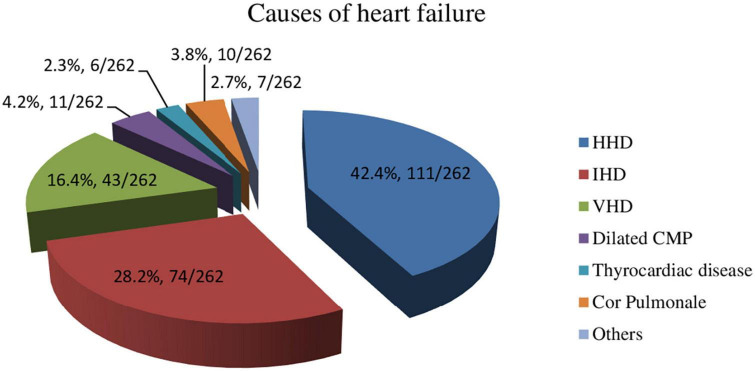
Causes of heart failure among patients aged 50 years or older with heart failure.

Hypertensive heart disease (111/262, 42%) was the most common cause of heart failure, followed by ischemic heart disease (74/262, 28%) and valvular heart disease (43/262, 16%) (Figure 1). Three-fourths (196/262, 75%) of the patients had cardiac disease for three or more years. Two-thirds (164/262, 63%) of the patients had shortness of breath and fatigue while performing ordinary activities (New York Heart Association (NYHA) functional class II). Two-thirds (171/262, 65%) of patients had associated comorbidities. Hypertension (111/171, 65%) was the frequently observed comorbid disease. Angiotensin-converting enzyme inhibitors (180/262, 69%), beta-blockers (157/262, 60%), diuretics (131/262, 50%), aldosterone antagonists (52/262, 20%), and digoxin (22/262, 8%) were the frequently prescribed cardiac medications (Tables 2, 3).

**TABLE 2:** Clinical characteristics of older adult patients with heart failure at Cardiac Clinic, University of Gondar hospital, June 01, 2021 to October 31, 2021 (No. 262).

Characteristics	Frequency (No.)	Percentage (%)
**Causes of heart failure**		
Hypertensive heart disease	111	42.4
Ischemic heart disease	74	28.2
Valvular heart disease	43	16.4
Dilated cardiomyopathy	11	4.2
Thyrocardiac disease	6	2.3
Cor Pulmonale	10	3.8
Others*	7	2.7
**Duration of heart failure**		
≤1 years	66	25.2
1–3 years	98	37.4
3–5 years	57	21.8
>5 years	41	15.6
**Severity of heart failure (NYHA)**		
NYHA I/II (Early disease)	204	77.9
NYHA III/IV (Advanced disease)	58	12.1
**Cardiac medications history**		
ACEIs (Enalapril)	180	68.7
Beta-Blockers (Metoprolol)	157	59.9
Diuretics (Furosemide)	131	50.0
Aldosterone antagonist (Spironolactone)	52	19.8
Digitalis glycosides (Digoxin)	22	8.4

*Congenital heart disease, pericardial disease.

**TABLE 3:** Co-morbidities of older adult patients with heart failure at Cardiac Clinic, University of Gondar hospital, June 01, 2021 to October 31, 2021 (No. 171).

Comorbidities type	Frequency	Percenatge (%)
Diabetes mellitus	12	7.0
Thyrotoxicosis	33	19.3
HTN*	111	64.9
Atrial fibrillation	44	25.7
Stroke	4	2.3
CKD*	3	1.8
COPD*	17	9.9
Others**	7	4.1

*HTN, hypertension; CKD, chronic kidney disease; COPD, chronic obstructive pulmonary disease. **Pulmonary tuberculosis, interstitial lung disease.

### Components of mini-nutritional assessment-full form score

Among a total of 262 patients, lower body mass index (BMI < 19 kg/m^2^), mid-arm circumference (MAC < 21 cm), and calf circumference (CC < 31 cm) were observed in 89/262 (34%), 86/262 (33%), and 135/262 (52%) of the study subjects, respectively (Table 4). Only one-fifth (47/262, 18%) of the study subjects suffered from exacerbation of heart failure in the last 3 months. Most (246/262, 94%) of them went out outdoors and lived independently (246/262, 94%). More than two-thirds (187/262, 71%) of patients were on three or more prescribed drugs (Table 5). More than half (147/262, 52%) of the study subjects did not know their weight loss status in the past 3 months. Two-fifths (103/262, 39%) of the patients had decreased food intake over the past 3 months. Almost all (257/262, 98%) patients got three meals per day. Most (252/262, 96%) of the study subjects got neither dairy products (milk, cheese, or yogurt) nor meat products (meat, fish, or poultry) (260/262, 99.2%) on a daily basis. Most (229/262, 87%) of the study subjects had two or more servings of vegetables per day. Almost all (258/262, 99%) of the study subjects self-fed themselves without any assistance (Table 6). More than half (135/262, 52%) of the study subjects self-perceived as uncertain about their nutritional status. Close to one-third (74/262, 28%) of the study subjects considered themselves to have poor health status as compared to their age matches (Table 7).

**TABLE 4:** Anthropometric assessment of older adult patients with heart failure at Cardiac Clinic, University of Gondar hospital, June 01, 2021 to October 31, 2021 (No. 262).

Characteristics	Category	Frequency (No.)	Percentage
**Mid arm circumference (cm)**			
	<21	86	32.8
	21–22	47	17.9
	>22	129	49.2
**Calf circumference (cm)**			
	<31	135	51.5
	>31	127	48.5
**BMI (kg/m^2^)**			
	<19	89	34.0
	19–21	55	21.0
	21–23	35	13.3
	>23	83	31.7
**Weight loss during the last 3 months**			
	Weight loss 1–3 kg	8	3.1
	Weight loss > 3kg	2	0.8
	Does not know	147	56.1
	No weight loss	105	40.1

BMI, body mass index; m, meter; cm, centimeter, Kg, kilogram.

**TABLE 5:** General assessment of older adult patients with heart failure at Cardiac Clinic, University of Gondar hospital, June 01, 2021 to October 31, 2021 (No. 262).

Characteristics	Category	Frequency (No.)	Percentage (%)
Mobility	Bed/chair bound Able to move out of bed/chair but does not go out Goes out	2 14 246	0.8 5.3 93.9
Lives independently	Yes No	246 16	93.9 6.1
Suffered psychological stress/acute illness in the past 3 months	Yes No	47 215	17.9 82.1
Neuropsychological problems	Severe dementia/depression Mild dementia No psychological problem	1 14 247	0.4 5.3 94.3
Pressure sore/Skin ulcer	Yes No	1 261	0.4 99.6
Takes more than 3 prescription	Yes No	187 75	71.4 28.6

**TABLE 6:** Dietary assessment in older adult patients with heart failure at Cardiac Clinic, University of Gondar hospital, June 01, 2021 to October 31, 2021 (No. 262).

Characteristics	Category	Frequency (No.)	Percentage (%)
Food intake decline over the past 3 months	Severe decrease in food intake Moderate decrease in food intake No decrease in food intake	3 100 159	1.1 38.2 60.7
How many full meals does the patient have per day	2 meals 3 meals	5 257	1.9 98.1
At least one serving of dairy products per day	Yes No	10 252	3.8 96.2
Meat, fish, poultry every day	Yes No	2 260	0.8 99.2
Two or more servings of fruit/vegetable PER Day	Yes No	229 33	87.4 12.6
How much fluid is consumed per day	Less than 3 cups 3–5 cups More than 5 cups	8 215 39	3.1 82.1 14.9
Mode of feeding	Unable to eat without	1	0.4
	Assistance A self-feed with some difficulty A self-feed without any Problem	3 258	1.1 98.5

**TABLE 7:** Self-assessment of older adult patients with heart failure at Cardiac Clinic, University of Gondar hospital, June 01, 2021 to October 31, 2021 (No. 262).

Characteristics	Category	Frequency (No.)	Percentage (%)
In comparison with other people of the same age consider his/her health status	Not as good Does not know As good Better	74 107 76 5	28.2 40.8 29.1 1.9
**Self-view of nutritional status**			
	View self as malnourished Uncertain of nutritional status View self as having no nutritional problem	8 135 119	3.1 51.5 45.4

### Mini-nutritional assessment-full form score

Based on the MNA-FL score, 75 out of 262 (28.6%, 95% CI: 22.9–34.4%) subjects were undernourished (MNA-FL < 17), while 124 out of 262 (47.3%, CI: 41.5–53.1%) were at risk of undernutrition (MNA-FL = 17–23.5). The remaining 63 out of 262 (24.1%, 95% CI: 18.2–29.8%) study subjects were well nourished (MNA-FL ≥ 24) (Table 8).

**TABLE 8:** Mini nutritional assessment (MNA) outcome of older adult patients with heart failure at Cardiac Clinic, University of Gondar hospital, June 01, 2021 to October 31, 2021.

Characteristics	Category	Frequency (No.)	Percentage (%)
MNA-FL score	<17 17–23.5 ≥24	75 124 63	28.62 47.33 24.05
Malnutrition (under nutrition)	Yes No	75 187	28.6 71.4

MNA-FL, mini nutritional assessment-full form.

### Factors associated with undernutrition in patients with heart failure

Bivariate logistic regression analysis revealed that those patients with severe heart failure (NYHA functional class III/IV), longer duration of illness, presence of comorbidities, treatment with loop diuretics and aldosterone antagonists, and rural residents were at risk of developing undernutrition. However, those treated with ACEIs were protected from undernutrition. When variables with a *P*-value of <0.25 in bivariate analysis were regressed further in multivariate analysis, patients with severe heart failure (AOR = 4.287, CI: 2.012–9.134, *P*-value < 0.001), with a duration of illness of 3–5 years (AOR = 3.225, CI: 1.138–9.137, *P*-value = 0.028), with a duration of illness of >5 years (AOR = 4.349, CI: 1.592–11.879, *P*-value = 0.001), with the presence of comorbidities (AOR = 2.29, CI: 1.06–4.96, *P*-value = 0.036), who underwent treatment with loop diuretics (AOR = 2.983, CI: 1.407–6.326, *P*-value = 0.040), and who were rural residents (AOR = 5.119, CI: 2.481–10.560, *P*-value < 0.001) were at risk of developing undernutrition (Table 9).

**TABLE 9:** Bivariate and multivariate analysis of older adult patients with heart failure at Cardiac Clinic, University of Gondar hospital, June 01, 2021 to October 31, 2021 (No. 262).

Variables	Under nutrition (MNA < 17)		Crude odds ratio	*P*-value	Adjusted odds ratio	*P*-value
Age (years)	Yes	No				
50–65	36	102	1		1	
66–75	21	57	0.596 (0.288, 1.231)	0.162	0.583 (0.240, 1.418)	0.234
>75	18	28	0.958 (0.511, 1.796)	0.893	0.771 (0.348, 1.707)	0.521
**Gender**						
Male	27	81	1			
Female	48	106	1.358 (0.781, 2.362)	0.278		
**Residency**						
Rural	60	79	5.468 (2.895, 10.328)	<0.001	5.119 (2.481, 10.560)	<0.001
Urban	15	108	1		1	
**Duration of heart failure**						
<1 years	17	49	1		1	
1–3 years	17	81	1.374 (0.6009, 3.098)	0.444	1.730 (0.664, 4.642)	0.277
3–5 years	22	35	2.489 (1.091, 5.682)	0.030	3.225 (1.138, 9.137)	0.028
>5 years	19	22	4.115 (1.837, 9.216)	0.001	4.349 (1.592, 11.879)	0.001
**Severity of heart failure (NYHA functional class)**						
NYHA class I/II	39	165	1			
NYHA class III/IV	36	22	6.923 (3.669, 13.063)	<0.001	4.287 (2.012, 9.134)	<0.001
**Co-morbidities**						
Yes	60	111	2.667 (1.409–5.045)	0.028	2.291 (1.062–4.960)	0.036
No	15	74	1			
**Cardiac medications history/type**						
**ACE inhibitors (Enalapril)**						
Yes	46	134	0.627 (0.357, 1.102)	0.105	0.631 (0.319, 1.148)	0.186
No	21	53	1			
**Beta-blockers (Metoprolol)**						
Yes	42	115	0.797 (0.463, 1.371)	0.412		
No	33	72	1			
**Diuretics (furosemide)**						
Yes	57	74	4.836 (2.639, 8.860)	0.010	2.983 (1.407, 6.326)	0.040
No	18	113	1		1	
**Aldosterone antagonists (Spironolactone)**						
Yes	24	28	2.672 (1.423, 5.017)	0.020	1.146 (0.506, 2.593)	0.074
No	51	159	1		1	
**Digitalis glycosides (digoxin)**						
Yes	8	14	1.475 (0.592, 3.678)	0.401		
No	67	173	1			

NYHA, new york neart association.

## Discussion

The magnitude of undernutrition among patients with heart failure was found to be 28.6% (95% CI: 22.9–34.4%). The estimated prevalence of undernutrition was 25–40% from an observational study by Rosa-Maria et al. (9). A recent meta-analysis by Lv et al. documented that the prevalence of undernutrition was 46% (95% CI: 43–49%) (10). It was reported to be 78% in previous studies in Ethiopia (19). The wide-ranging magnitude of the estimated prevalence of undernutrition among patients with heart failure could be explained by the difference in the type of nutritional risk assessment tools used, patient-related characteristics, severity of heart disease, and coexisting comorbidities. Heart failure is significantly associated with protein-calorie undernutrition. Undernutrition in heart failure is triggered by poor appetite and nutritional intake, malabsorption from the gut wall and mucosal abnormalities, hypercytokinemia from systemic inflammation, neurohumoral and immunologic activation, oxidative stress, and excessive resting energy expenditure (REE) from an imbalance in the anabolic–catabolic process (4–7). Cardiac wasting and cachexia are associated with frequent symptom exacerbations, higher hospital readmission rates, longer hospital stay, poor quality of life, and increased mortality rates (7–11). The nutritional intervention had shown to reduce hospital readmission rate and mortality rates in undernourished patients with heart failure (11, 12, 14–16). Increased lean body mass and fat tissue mass after dietary interventions were linked to an increase in the quality of life and better survival, respectively (11, 16, 17). Neurohumoral blockade with ACEIs and beta-blockers showed a survival benefit, improved quality of life, and prevented unintentional weight loss or induced edema-free weight gain (5, 11, 12). Appetite stimulators and anabolic steroids were proven to increase body mass in patients with heart failure in a few clinical trials. However, the harm outweighed the benefit of anti-inflammatory and immunomodulatory drug use. Selective intestinal decontamination to modulate intestinal microflora was not found to be beneficial (4, 5, 11, 16, 17). Hypertensive heart disease accounted for nearly half (42%) of the causes of heart failure. The finding (39%) was congruent with the sub-Saharan African systematic review by Agbor et al. (31). Hypertension (65%) was the commonest comorbid disease. Primary data from Amare et al. and systemic review by Agbor VN, et al. documented that hypertension was the frequently observed (36–39%) comorbid disease (18, 31). On multivariate logistic regression analysis, severe heart failure, longer duration of cardiac disease, coexisting comorbidities, use of loop diuretics, and rural residence were significantly associated with undernutrition in patients with heart failure. Patients with severe heart failure (NYHA functional class III/IV) were four times more at risk of developing undernutrition as compared to those with mild heart failure (NYHA functional class I/II) (AOR = 4.287, CI: 2.012–9.134, *P*-value < 0.001). A review by Dunn et al. documented that there was an increased incidence of nutritional wasting with the severity of heart failure, ranging from 22% in heart failure NYHA class II to 63% in heart failure NYHA class III (11). Similarly, exacerbated nutritional wasting was observed among patients with advanced heart failure from a report by Kaluzna-Oleksy et al. (12). A study by Rosa-Maria et al. recognized that undernutrition was a mediator of disease progression and determining prognosis in advanced heart failure (10). Patients with a longer duration of cardiac illness (≥3 years) were three to four times more likely to develop undernutrition as compared to those with a shorter duration of cardiac illness (<3 years). Various studies showed that chronicity in heart failure was associated with maladaptive neurohumoral activation, hypercytokinemia, and hypercatabolic state, which resulted in malnutrition–inflammation–cachexia (MIC) (4–6, 14). The odds of developing undernutrition were 2-fold higher in those who had comorbidities as compared to those who had not (AOR = 2.29, CI: 1.06–4.96, *P*-value = 0.036). The identified comorbidities among study subjects were known to cause hypercytokinemia from systemic inflammation and excessive REE from an increased catabolic state. The odds of developing undernutrition were three times higher in patients who were on diuretic therapy as compared to those who were not (AOR = 2.983, CI: 1.407–6.326, *P*-value = 0.040). It could be partly explained by diuretic-induced excessive renal loss of micronutrients and trace elements, or those patients on diuretic treatment might have advanced heart failure (11). Patients from rural areas were five times more at risk of developing undernutrition as compared to urban dwellers (AOR = 5.119, CI: 2.481–10.560, *P*-value < 0.001). This finding might be explained by differences in dietary habits and socioeconomic conditions among urban and rural dwellers in the catchment area. A study done in South Africa documented that urbanization was associated with an increased intake of fat, meat, sugar, and beverages. Rural residents often consumed traditional foodstuffs and had limited food choices (32).

### Limitations of the study

The cross-sectional study design might have a limitation on causal relationships and the absence of a control arm. The study subjects were relatively older adult patients, who might not be a true representative of the catchment population. In addition, the convenience sampling method was used, which might introduce selection bias.

## Conclusion

Undernutrition was a significant clinical problem in older adult patients with heart failure. Chronic and severe heart failure, presence of comorbidities, treatment with diuretics, and rural residence were significant predictors of undernutrition in patients with heart failure.

## Data availability statement

The raw data supporting the conclusions of this article will be made available by the authors, without undue reservation.

## Ethics statement

The studies involving human participants were reviewed and approved by Institutional Review Board (IRB) of the College of Medicine and Health Sciences, University of Gondar (02/08/2021, IRB No. 755/2021). The patients/participants provided their written informed consent to participate in this study.

## Author contributions

HuA contributed to the conception, design, data collection, analysis, writing, and review of the manuscript. AT contributed to the conception, design, analysis, writing, and review of the manuscript. HaA, AA, and MT contributed to the conception, design, analysis, and review of the manuscript. All authors read and approved the final manuscript and approved its submission for publication.

## Abbreviations

ACCF: American College of Cardiology Foundation
ACEIs: angiotensin converting enzyme inhibitors
AHA: American Heart Association
AOR: adjusted odds ratio
BMI: body mass index
CABG: coronary artery bypass graft
CC: calf circumference
CHF: congestive heart failure
CI: confidence interval
COR: crude odds ratio
Cm: centimeter
Echo: echocardiography
ECG: electrocardiography
IRB: Institutional Review Board
Kg: kilogram
M: meter
MAC: mid-arm circumference
MIC: malnutrition-inflammation-cachexia
MNA-FL: mini-nutritional assessment-full form
NYHA: New York Heart Association
PCI: percutaneous coronary intervention
REE: resting energy expenditure
SD: standard deviation.
